# Association between COVID-19 vaccine hesitancy and generalized trust, depression, generalized anxiety, and fear of COVID-19

**DOI:** 10.1186/s12889-021-12479-w

**Published:** 2022-01-18

**Authors:** Yoichi Sekizawa, Sora Hashimoto, Kenzo Denda, Sae Ochi, Mirai So

**Affiliations:** 1 Research Institute of Economy, Trade and Industry, Chiyoda-ku, Kasumigaseki 1-3-1, Tokyo, 100-8901 Japan; 2 United Health Communication Co., Ltd., Chuo-ku, Nihonbashitomizawacho 10-16, Tokyo, 103-0006 Japan; 3 Hiramatsu Memorial Hospital, Chuo-ku Minami 22-jo nishi 14-1-20, Sapporo-shi, Hokkaido 064-8536 Japan; 4 Department of Laboratory Medicine, The Jikei University School of Medicine, Minato-ku Nishishinbashi 3-25-8, Tokyo, 105-8461 Japan; 5 Department of Psychiatry, Tokyo Dental College Ichikawa General Hospital, Ichikawa-shi, Sugano 5-11-13, Chiba, 272-8513 Japan

**Keywords:** COVID-19, Vaccine hesitancy, Generalized trust, Depression, Generalized anxiety, Fear of COVID-19

## Abstract

**Background:**

Although numerous studies have been published on the predictors of COVID-19 vaccine hesitancy, some possible predictors remain underexplored. In this study, we explored the associations of unwillingness and indecisiveness regarding COVID-19 vaccination with generalized trust, mental health conditions such as depression and generalized anxiety, and fear of COVID-19.

**Methods:**

Data of wave 1 (from October 27 till November 6, 2020) and wave 3 (from April 23 till May 6, 2021) of a longitudinal online study conducted in Japan were used for the analyses. Unvaccinated participants were asked at wave 3 about their willingness to be vaccinated, with possible responses of willing, unwilling, or undecided. These three responses were used as the outcome variable, and multinomial logistic regression analyses were conducted with willingness to be vaccinated as the reference group. Explanatory variables included generalized trust, depression, generalized anxiety, and fear of COVID-19 both at wave 1 and 3, and sociodemographic and health-related variables.

**Results:**

Of the 11,846 valid respondents, 209 (1.8%) answered that they had already been vaccinated against COVID-19, 7089 (59.8%) responded that they were willing to be vaccinated, 3498 (29.5%) responded that they were undecided, and 1053 (8.9%) responded that they were unwilling to be vaccinated. After adjusting for covariates, we found that: (1) participants with lower levels of generalized trust at wave 1 and 3 were more likely to be undecided or unwilling at wave 3; (2) respondents with moderately severe or severe depression at wave 1 and 3 were more likely to be undecided at wave 3; (3) participants with moderate or severe levels of generalized anxiety at wave 3 but not at wave 1 were more likely to be unwilling at wave 3; and (4) respondents with high levels of fear of COVID-19 at wave 1 and 3 were less likely to be undecided and unwilling at wave 3.

**Conclusions:**

Generalized trust, mental health conditions such as depression and generalized anxiety, and low level of fear of COVID-19 are associated with unwillingness or indecision regarding being vaccinated against COVID-19.

**Supplementary Information:**

The online version contains supplementary material available at 10.1186/s12889-021-12479-w.

## Background

COVID-19 has been prevalent around the world since the end of 2019, and many people have lost their lives due to this infectious disease. Many countries have implemented strict measures to control the spread of the disease, such as lockdowns and mandatory wearing of face masks; despite these measures, the disease has spread across the world.

Vaccination is one of the most effective ways of halting the spread of COVID-19. Several vaccines have been developed over a short period of time. Vaccination not only helps prevent individual infections, but mass vaccination prevents medical resources from being overwhelmed by lowering the number of critically ill patients and has the potential to halt the pandemic through the achievement of herd immunity. As of November 27, 2021, almost half of the global population has received at least one dose of the COVID-19 vaccine, and more than 7 billion doses have been administered worldwide [1].

Vaccine hesitancy has been defined as a “delay in acceptance or refusal of vaccination despite availability of vaccination services” [2]. It creates a serious problem in the promotion of vaccination against COVID-19 [3]. Despite the importance of vaccination, many people are known to have a negative attitude toward it [4]. There is a concern that even if a vaccination program progresses initially, it may stall at some point because of the large number of people in many countries who refuse to be vaccinated [5]. An exploration of the type of people who are less likely to receive a vaccine against COVID-19 is important in order to cover maximum range of people with vaccination. Numerous studies that address the question of which people were reluctant to be vaccinated have been published recently [6–23]. Most of these studies showed that younger people [7, 8, 12, 14–16, 19–23], women [6, 7, 9–12, 14–16, 19–23], people with low incomes [6, 11, 13–15, 18, 19, 21, 22], and people with lower levels of education [6, 7, 10, 11, 13–16, 18–23] were reluctant to receive a vaccine.

Although many predictors of vaccine hesitancy have been identified, some possible predictors remain underexplored. Of these, we examined the associations between vaccination hesitancy and generalized trust, mental health factors such as depression and generalized anxiety, and fear of COVID-19.

Specific trust, such as trust in vaccines, the government, the health system, or professionals, has been sufficiently explored in the context of vaccine hesitancy in general [24, 25], and specifically for COVID-19 vaccines (trust in vaccines [6, 16, 26–28], trust in government [20, 22, 26, 29, 30], trust in healthcare system or practitioners [16, 31–33]). By contrast, generalized trust, i.e., a default expectation of goodwill from other people [34] that is often viewed as an important part of social capital [35], has not been sufficiently explored in the context of vaccine hesitancy. We are aware of two studies that focus on the association between generalized trust and COVID-19: Frank and Arim [36] and Ahorsu et al. [31]. Both reported that those who indicated higher levels of generalized trust tended to be more willing to receive the COVID-19 vaccine than those with lower levels of generalized trust. Regarding the association between generalized trust and vaccine hesitancy other than for COVID-19, we found three studies, and all of them concluded that generalized trust was associated with higher willingness to receive a vaccine against influenza [37–39].

Similar to generalized trust, studies that explore the attitudes of people with mental illness toward vaccination against COVID-19 are scarce [40] and inconsistent. To our knowledge, there have been five studies in the United States, China, Germany, and Japan [15, 17, 22, 41, 42]. Three of them found no significant association between depression/anxiety and vaccine hesitancy [15, 17, 41]. By contrast, a study in China that concentrated on people with children found that psychologically distressed subjects were more likely to have vaccine hesitancy related to themselves, their spouses, and their children [42]. Similarly, a study in Japan showed that respondents with severe psychological distress were more likely to have vaccine hesitancy related to the COVID-19 vaccines [22]. More broadly, studies that explore the relationship between depression/anxiety and vaccine hesitancy in general suggest that those who have depression/anxiety tend to be willing to be vaccinated mostly for influenza [43–45].

Regarding fear of COVID-19, several studies have already explored the association between fear of COVID-19 and vaccine hesitancy [15, 22, 26, 27, 41, 46–48]. All of them except one [46] found that fear of COVID-19 was positively associated with willingness to be vaccinated against COVID-19. More broadly, studies that explored the relationship between the willingness to receive vaccination and the fear of the disease the vaccine prevents concluded that those who fear the disease tend to be more willing to be vaccinated [49–53]. Nevertheless, a recent model study on the COVID-19 vaccine suggested that the impact of the fear of COVID-19 on the willingness to receive vaccination is small, although it exists [54]. Against this background, it is worthwhile to confirm the robustness of the association between fear of COVID-19 and the willingness to receive vaccination in a country like Japan where the prevalence of COVID-19 cases was lower than in most other countries [1].

In the present study, we explored two things. First, we explored whether generalized trust, mental health factors such as depression and generalized anxiety, and fear of COVID-19 were predictors of COVID-19 vaccine hesitancy. Second, we explored whether the results of the previous studies on predictors, in particular socioeconomic ones, of vaccine hesitancy against COVID-19 can be confirmed with a large data of Japanese people.

## Methods

### Research design, participants, and procedures

A longitudinal online survey named “Continuing survey on mental and physical health during the COVID-19 pandemic” (hereinafter “RIETI questionnaire survey”) was planned and organized by the authors as part of a research project of the Research Institute of Economy, Trade and Industry, Japan (RIETI). This longitudinal survey was conducted by NTTCom Online Marketing Solutions Corporation based on the design and instructions of the authors. We used wave 1 and wave 3 of the RIETI questionnaire survey for the present study. Wave 1 was conducted from October 27 to November 6, 2020. Wave 3 was conducted from April 23 to May 6, 2021. Study participants were recruited from the registrants of the corporation or its affiliates. The participants at wave 1 were men and women aged 18–74 years living in Japan. They were extracted so that their demographic composition ratios of sex, age, and distribution of residential prefectures matched the population estimates of the Statistics Bureau of Japan (final estimates, May 2020). Data were excluded for individuals who provided non-existent zip codes, zip codes that did not match their previously registered prefectures, extreme outlying values for height and weight among Japanese adults (2 m or taller and weight lower than 35 kg or in excess of 100 kg, measurements that are outside the national average in Japan), or age differing by 2 years or more from the age previously registered. Respondents who took a very short time (less than 5 min) or a very long time (10 h or more) to answer the survey questions were also excluded. The remaining number of individuals after these exclusions was 16,642 (8022 men, 8620 women) and they were established as valid respondents for wave 1. In the subsequent waves, we asked all valid respondents from wave 1 to respond to the surveys by email. For wave 3, those who took a very short time (less than 4 min) or a very long time (10 h or more) to answer the survey questions were excluded. All communication with study participants, including the questionnaires, was conducted in Japanese.

All individuals who participated in this study provided online informed consent for their participation. The present study was conducted with the approval of the ethics committee of Hiramatsu Memorial Hospital (No: 20200925). All methods were carried out in accordance with relevant guidelines and regulations.

### Outcome variables

The outcome variables used in the present study were based on the following two questions regarding vaccination against COVID-19, which were asked only in wave 3. The first question asked whether the respondent had been vaccinated. If participants had received at least one dose, they were treated as having been vaccinated. The second question was asked only of those who had not been vaccinated. The question was: “Are you going to receive a vaccine against COVID-19?” It was scored as willing to be vaccinated, unwilling to be vaccinated, or undecided.

### Explanatory variables

#### Generalized trust, depression, generalized anxiety, and fear of COVID-19

To measure generalized trust, we used the Japanese version of the following question from the World Values Survey (WVS) Wave 6 [55]: “Generally speaking, would you say that most people can be trusted or that you need to be very careful in dealing with people?” The available answers were as follows: “Most people can be trusted,” “Need to be very careful,” and “Don’t know.” Respondents who selected “Need to be very careful” and “Don’t know” were regarded as having low generalized trust. This question has been validated by two experimental studies [56, 57].

Depression was measured using the Patient Health Questionnaire-9 (PHQ-9) [58]. The PHQ-9 is a nine-item self-administered questionnaire designed to assess the presence and severity of depressive symptoms during the past 2 weeks. Example items include “little interest or pleasure in doing things” and “feeling down, depressed, or hopeless.” The answers in the PHQ-9 consist of a four-point scale ranging from 0 (not at all) to 3 (nearly every day). The PHQ-9 scores range from 0 to 27, with higher scores indicating progressively more severe depression (0–4 = no depression, 5–9 = mild depression, 10–14 = moderate depression, 15–19 = moderately severe depression, and 20–27 = severe depression). Although the original version of the PHQ-9 is in English, a Japanese version was used in the present study [59]. The Japanese version of PHQ-9 has been validated by Muramatsu et al. [60].

Anxiety was measured by means of the Generalized Anxiety Disorder-7 scale (GAD-7) [61]. The GAD-7 is a seven-item, self-administered questionnaire that is designed to measure generalized anxiety symptoms during the past 2 weeks. Example items include “feeling nervous, anxious, or on edge” and “not being able to stop or control worrying.” The answers in the GAD-7 consist of a four-point scale ranging from 0 (not at all) to 3 (nearly every day). The GAD-7 scores range from 0 to 21, with higher scores indicating progressively greater levels of anxiety (0–4 = minimal anxiety, 5–9 = mild anxiety, 10–14 = moderate anxiety, and 15–21 = severe anxiety). Although the original version of the GAD-7 is in English, a Japanese version was used in the present study [62]. The Japanese version of GAD-7 has been validated by its translators [62].

The Fear of COVID-19 Scale (FCV-19S) was used to measure fear related to COVID-19 [63]. The FCV-19S is a seven-item, self-reporting questionnaire designed to measure the extent to which a person fears COVID-19. Example items included “I am most afraid of coronavirus-19” and “it makes me uncomfortable to think about coronavirus-19.” The answers in the FCV-19S consist of a five-point scale ranging from 1 (strongly disagree) to 5 (strongly agree). The FCV-19S scores range from 7 to 35, with higher scores indicating progressively greater levels of fear related to COVID-19. Since there were no clear categories for the FCV-19S scores, we defined them as follows: 7–15 = no fear, 16–20 = mild fear, 21–25 = moderate fear, and 26–35 = severe fear. Although the original version of the FCV-19S is in English, a Japanese version was used in the present study [64]. The Japanese version of FCV-19S has been validated by its translators [64].

#### Other explanatory variables

Sociodemographic variables included sex, age, level of education, family members living together, employment status, annual household income, bank and saving deposit amount, and place of residence (individual prefectures with a population of 5 million or more [nine prefectures], and eight regions of Japan for those living in other prefectures). Marital status was not included because the question regarding family members living together was similar. For variables relating to health, we used body mass index (BMI) and whether pre-existing conditions (hypertension, dyslipidemia, diabetes, heart disease, renal disease, cancer, respiratory disease, and other) were present. Descriptions of the explanatory variables are shown with the basic statistics in Table 1.

**Table 1:** Characteristics and vaccine-related attitudes of study participants

Predictors	Already vaccinated	Not vaccinated			Total
		Willing	Undecided	Unwilling	
Overall sample	209 (1.8%)	7086 (59.8%)	3498 (29.5%)	1053 (8.9%)	11,846
Sex					
Male	102 (1.7%)	3778 (63.3%)	1583 (26.5%)	502 (8.4%)	5965
Female	107 (1.8%)	3308 (56.2%)	1915 (32.6%)	551 (9.4%)	5881
Age group, years at wave 1					
65+	28 (0.9%)	2377 (80.1%)	422 (14.2%)	142 (4.8%)	2969
50–64	72 (1.7%)	2696 (61.8%)	1262 (28.9%)	330 (7.6%)	4360
30–49	77 (2.2%)	1615 (46.9%)	1347 (39.1%)	402 (11.7%)	3441
18–29	32 (3.0%)	398 (37.0%)	467 (43.4%)	179 (16.6%)	1076
Age group, years at wave 3					
65+	30 (1.0%)	2482 (79.8%)	451 (14.5%)	148 (4.8%)	3111
50–64	74 (1.7%)	2671 (60.9%)	1296 (29.5%)	345 (7.9%)	4386
30–49	78 (2.3%)	1573 (46.6%)	1330 (39.4%)	394 (11.7%)	3375
18–29	27 (2.8%)	360 (37.0%)	421 (43.2%)	166 (17.0%)	974
Highest education					
Junior/senior high school	30 (0.8%)	2171 (57.2%)	1215 (32.0%)	378 (10.0%)	3794
Two- or three-year college	67 (2.6%)	1446 (57.0%)	779 (30.7%)	245 (9.7%)	2537
Four-year college or higher	112 (2.0%)	3469 (62.9%)	1504 (27.3%)	430 (7.8%)	5515
Family members living together at wave 1					
Living alone	40 (2.0%)	1043 (52.7%)	657 (33.2%)	241 (12.2%)	1981
Living only with spouse	45 (1.3%)	2479 (71.3%)	757 (21.8%)	198 (5.7%)	3479
Living with children	79 (2.2%)	2181 (60.8%)	1056 (29.4%)	274 (7.6%)	3590
Living with parents	28 (1.4%)	972 (48.3%)	756 (37.6%)	256 (12.7%)	2012
Three-generation household	14 (2.3%)	324 (54.4%)	205 (34.4%)	53 (8.9%)	596
Other (siblings only, friends, grandparents and grandchildren etc.)	3 (1.6%)	87 (46.3%)	67 (35.6%)	31 (16.5%)	188
Employment at wave 1					
Employed	184 (2.6%)	4043 (57.5%)	2163 (30.7%)	646 (9.2%)	7036
Homemaker	11 (0.5%)	1491 (64.8%)	659 (28.6%)	141 (6.1%)	2302
Not employed (seeking a job)	3 (1.0%)	140 (47.9%)	109 (37.3%)	40 (13.7%)	292
Not employed (not seeking a job)	6 (0.3%)	1250 (68.6%)	399 (21.9%)	166 (9.1%)	1821
Student	5 (1.9%)	98 (37.1%)	121 (45.8%)	40 (15.2%)	264
Other	0 (0.0%)	64 (48.9%)	47 (35.9%)	20 (15.3%)	131
Employment at wave 3					
Employed	185 (2.6%)	4023 (57.1%)	2199 (31.2%)	639 (9.1%)	7046
Homemaker	13 (0.6%)	1475 (65.0%)	646 (28.5%)	134 (5.9%)	2268
Not employed (seeking a job)	2 (0.6%)	159 (51.1%)	101 (32.5%)	49 (15.8%)	311
Not employed (not seeking a job)	7 (0.4%)	1278 (68.4%)	407 (21.8%)	176 (9.4%)	1868
Student	2 (0.9%)	85 (39.4%)	97 (44.9%)	32 (14.8%)	216
Other	0 (0.0%)	66 (48.2%)	48 (35.0%)	23 (16.8%)	137
Annual household income, million yen at wave 1					
< 3	35 (1.2%)	1611 (53.2%)	1013 (33.4%)	372 (12.3%)	3031
3–4	42 (1.3%)	2071 (63.6%)	872 (26.8%)	269 (8.3%)	3254
5–7	68 (2.2%)	1887 (59.7%)	981 (31.0%)	225 (7.1%)	3161
≥ 8	64 (2.7%)	1517 (63.2%)	632 (26.3%)	187 (7.8%)	2400
Annual household income, million yen at wave 3					
< 3	32 (1.0%)	1680 (54.3%)	1008 (32.6%)	372 (12.0%)	3092
3–4	45 (1.4%)	2024 (62.8%)	888 (27.5%)	267 (8.3%)	3224
5–7	67 (2.1%)	1885 (59.6%)	988 (31.2%)	224 (7.1%)	3164
≥ 8	65 (2.7%)	1497 (63.3%)	614 (26.0%)	190 (8.0%)	2366
Bank and savings deposit amount, million yen at wave 1					
< 1	63 (2.0%)	1529 (49.5%)	1121 (36.3%)	373 (12.1%)	3086
1–3	39 (1.7%)	1286 (56.5%)	743 (32.6%)	209 (9.2%)	2277
4–9	47 (2.0%)	1421 (60.0%)	706 (29.8%)	196 (8.3%)	2370
≥ 10	60 (1.5%)	2850 (69.3%)	928 (22.6%)	275 (6.7%)	4113
Bank and savings deposit amount, million yen at wave 3					
< 1	51 (1.6%)	1538 (49.1%)	1153 (36.8%)	390 (12.5%)	3132
1–3	51 (2.3%)	1258 (56.8%)	714 (32.2%)	191 (8.6%)	2214
4–9	39 (1.7%)	1417 (61.1%)	687 (29.6%)	176 (7.6%)	2319
≥ 10	68 (1.6%)	2873 (68.7%)	944 (22.6%)	296 (7.1%)	4181
BMI at wave 1					
< 18.5	23 (1.6%)	739 (50.4%)	524 (35.7%)	181 (12.3%)	1467
18.5–24.9	148 (1.8%)	4930 (60.5%)	2377 (29.2%)	693 (8.5%)	8148
25.0–29.9	32 (1.7%)	1210 (64.3%)	487 (25.9%)	153 (8.1%)	1882
≥ 30.0	6 (1.7%)	207 (59.3%)	110 (31.5%)	26 (7.4%)	349
Pre-existing condition at wave 1					
Hypertension	34 (1.6%)	1577 (74.8%)	402 (19.1%)	96 (4.6%)	2109
Dyslipidemia	25 (2.1%)	910 (76.2%)	200 (16.8%)	59 (4.9%)	1194
Diabetes	9 (1.4%)	482 (72.5%)	136 (20.5%)	38 (5.7%)	665
Heart disease	7 (2.2%)	234 (74.3%)	56 (17.8%)	18 (5.7%)	315
Renal disease	2 (2.1%)	66 (68.8%)	23 (24.0%)	5 (5.2%)	96
Cancer	4 (2.0%)	146 (73.7%)	36 (18.2%)	12 (6.1%)	198
Respiratory disease	5 (1.8%)	180 (65.2%)	66 (23.9%)	25 (9.1%)	276
Other	2 (1.2%)	102 (60.0%)	50 (29.4%)	16 (9.4%)	170
Generalized trust at wave 1					
Most people can be trusted	70 (1.8%)	2604 (67.9%)	929 (24.2%)	234 (6.1%)	3837
Need to be very careful	132 (1.8%)	4220 (56.4%)	2369 (31.7%)	760 (10.2%)	7481
Don’t know	7 (1.3%)	262 (49.6%)	200 (37.9%)	59 (11.2%)	528
Generalized trust at wave 3					
Most people can be trusted	63 (1.7%)	2532 (69.5%)	822 (22.6%)	225 (6.2%)	3642
Need to be very careful	139 (1.8%)	4287 (55.9%)	2473 (32.3%)	765 (10.0%)	7664
Don’t know	7 (1.3%)	267 (49.4%)	203 (37.6%)	63 (11.7%)	540
Depression (PHQ-9) at wave 1					
None (0–4)	113 (1.5%)	4761 (65.1%)	1899 (26.0%)	540 (7.4%)	7313
Mild (5–9)	56 (2.0%)	1533 (54.2%)	949 (33.6%)	290 (10.3%)	2828
Moderate (10–14)	24 (2.4%)	497 (49.2%)	375 (37.1%)	115 (11.4%)	1011
Moderately severe (15–19)	12 (2.5%)	209 (44.0%)	183 (38.5%)	71 (14.9%)	475
Severe (20–27)	4 (1.8%)	86 (39.3%)	92 (42.0%)	37 (16.9%)	219
Depression (PHQ-9) at wave 3					
None (0–4)	110 (1.5%)	4784 (65.0%)	1918 (26.1%)	545 (7.4%)	7357
Mild (5–9)	51 (1.9%)	1511 (55.2%)	919 (33.6%)	257 (9.4%)	2738
Moderate (10–14)	29 (2.9%)	487 (48.7%)	352 (35.2%)	131 (13.1%)	999
Moderately severe (15–19)	10 (2.1%)	209 (43.1%)	192 (39.6%)	74 (15.3%)	485
Severe (20–27)	9 (3.4%)	95 (35.6%)	117 (43.8%)	46 (17.2%)	267
Generalized anxiety (GAD-7) at wave 1					
Minimal (0–4)	140 (1.6%)	5628 (63.0%)	2453 (27.5%)	714 (8.0%)	8935
Mild (5–9)	38 (2.0%)	998 (52.8%)	662 (35.0%)	192 (10.2%)	1890
Moderate (10–14)	23 (3.3%)	321 (46.0%)	260 (37.2%)	94 (13.5%)	698
Severe (15–21)	8 (2.5%)	139 (43.0%)	123 (38.1%)	53 (16.4%)	323
Generalized anxiety (GAD-7) at wave 3					
Minimal (0–4)	133 (1.5%)	5588 (63.3%)	2412 (27.3%)	690 (7.8%)	8823
Mild (5–9)	43 (2.2%)	1032 (52.5%)	694 (35.3%)	197 (10.0%)	1966
Moderate (10–14)	22 (3.4%)	306 (47.1%)	226 (34.8%)	96 (14.8%)	650
Severe (15–21)	11 (2.7%)	160 (39.3%)	166 (40.8%)	70 (17.2%)	407
Fear of COVID-19 (FCV-19S) at wave 1					
No fear (7–15)	65 (1.8%)	2066 (56.7%)	1056 (29.0%)	456 (12.5%)	3643
Mild (16–20)	79 (1.9%)	2556 (62.8%)	1152 (28.3%)	285 (7.0%)	4072
Moderate (21–25)	45 (1.5%)	1761 (59.5%)	934 (31.6%)	218 (7.4%)	2958
Severe (26–35)	20 (1.7%)	703 (59.9%)	356 (30.3%)	94 (8.0%)	1173
Fear of COVID-19 (FCV-19S) At wave 3					
No fear (7–15)	55 (1.8%)	1686 (54.7%)	869 (28.2%)	472 (15.3%)	3082
Mild (16–20)	72 (1.8%)	2514 (62.7%)	1159 (28.9%)	262 (6.5%)	4007
Moderate (21–25)	56 (1.7%)	1992 (60.1%)	1041 (31.4%)	228 (6.9%)	3317
Severe (26–35)	26 (1.8%)	894 (62.1%)	429 (29.8%)	91 (6.3%)	1440
Region of residence at wave 1					
Tokyo	21 (1.7%)	718 (57.9%)	368 (29.7%)	133 (10.7%)	1240
Kanagawa	9 (1.0%)	528 (61.5%)	266 (31.0%)	56 (6.5%)	859
Osaka	10 (1.2%)	520 (61.3%)	240 (28.3%)	78 (9.2%)	848
Aichi	10 (1.4%)	449 (61.8%)	203 (28.0%)	64 (8.8%)	726
Saitama	9 (1.3%)	413 (59.8%)	220 (31.8%)	49 (7.1%)	691
Chiba	8 (1.3%)	374 (60.9%)	165 (26.9%)	67 (10.9%)	614
Hyogo	3 (0.5%)	341 (62.1%)	153 (27.9%)	52 (9.5%)	549
Hokkaido	7 (1.3%)	328 (61.9%)	146 (27.5%)	49 (9.2%)	530
Fukuoka	5 (1.1%)	259 (59.3%)	120 (27.5%)	53 (12.1%)	437
Tohoku region	14 (1.7%)	509 (63.1%)	220 (27.3%)	64 (7.9%)	807
North Kanto	14 (2.3%)	341 (56.7%)	195 (32.4%)	51 (8.5%)	601
Hokuriku	9 (1.9%)	280 (60.6%)	138 (29.9%)	35 (7.6%)	462
Chubu	16 (1.9%)	492 (59.3%)	260 (31.4%)	61 (7.4%)	829
Kinki	15 (1.8%)	513 (60.1%)	240 (28.1%)	85 (10.0%)	853
Chugoku	19 (2.8%)	384 (55.8%)	230 (33.4%)	55 (8.0%)	688
Shikoku	12 (3.5%)	214 (62.4%)	87 (25.4%)	30 (8.7%)	343
Kyushu and Okinawa	28 (3.6%)	423 (55.0%)	247 (32.1%)	71 (9.2%)	769

### Statistical analysis

Descriptive statistics were presented with frequency and percentages for categorical variables. Continuous variables were grouped into categorical variables (e.g., age, BMI, household income). Multinomial logistic regression analyses were conducted to estimate the associations between the explanatory variables and the outcome variables. Respondents willing to receive a vaccine at wave 3 (reference group) were compared with (1) those who were undecided regarding vaccination; and (2) those who were unwilling to be vaccinated. We estimated four models. In Model 1 at wave 1 and Model 1 at wave 3, the explanatory variables of primary interest were any one of generalized trust, depression (PHQ-9), generalized anxiety (GAD-7), and fear of COVID-19 (FCV-19S) experienced during wave 1 and wave 3, respectively. In Model 2 at wave 1 and Model 2 at wave 3, the explanatory variables of primary interest were all of generalized trust, PHQ-9, GAD-7, and FCV-19S together, at wave 1 and wave 3, respectively. All other explanatory variables were adjusted for in all models. Generalized trust, PHQ-9, GAD-7, FCV-19S, age, employment status, annual household income, and bank and saving deposit amounts in Models 1 and 2 at wave 1 and wave 3 were those at wave 1 and 3, respectively. Remaining explanatory variables were those at wave 1. For all models we calculated the relative risk ratio (RRR) and the 95% confidence interval. Statistical analyses were carried out using STATA 15.0 (STATACorp LLC, Texas, USA).

## Results

### Study participants and characteristics

In wave 3, we sent surveys via email to the 16,642 valid respondents from wave 1, and the total number of responses was 13,279 (79.8%). After excluding those who took a very short time (less than 4 min) or a very long time (10 h or more) to answer the survey questions, the number of valid respondents was 11,846 (5965 males and 5881 females), and the mean age was 54.0 years (*SD =* 14.2 years). The basic statistics for the outcome variables and all explanatory variables are presented in Table 1.

### Attitude toward vaccination

Of the 11,846 valid respondents, 209 (1.8%) answered that they had already been vaccinated against COVID-19, 7089 (59.8%) responded that they were willing to be vaccinated, 3498 (29.5%) responded that they were undecided, and 1053 (8.9%) responded that they were unwilling to be vaccinated (Table 1).

### Association of generalized trust, depression, generalized anxiety, and fear of COVID-19 with attitudes toward vaccination against COVID-19

Table 2 presents the results of the adjusted multinomial logistic regression analyses focusing on the association of generalized trust, depression, generalized anxiety, and fear of COVID-19, with attitudes toward vaccination against COVID-19.

**Table 2:** Results of the adjusted multinomial logistic regression analyses

Dependent variable	Model 1 at wave 1			Model 1 at wave 3			Model 2 at wave 1			Model 2 at wave 3		
	RRR	95% CI	*P*-Value	RRR	95% CI	*P*-Value	RRR	95% CI	*P*-Value	RRR	95% CI	*P*-Value
Undecided on vaccination at wave 3												
Generalized trust												
Most people can be trusted	reference			reference			reference			reference		
Need to be very careful	**1.29**	(1.17–1.42)	< 0.001	**1.48**	(1.34–1.63)	< 0.001	**1.28**	(1.16–1.41)	< 0.001	**1.45**	(1.32–1.61)	< 0.001
Don’t know	**1.68**	(1.36–2.08)	< 0.001	**1.78**	(1.44–2.20)	< 0.001	**1.65**	(1.34–2.04)	< 0.001	**1.75**	(1.42–2.16)	< 0.001
Depression (PHQ-9)												
None (0–4)	reference			reference			reference			reference		
Mild (5–9)	**1.19**	(1.07–1.32)	< 0.001	**1.15**	(1.03–1.27)	0.010	**1.19**	(1.06–1.34)	0.003	1.10	(0.98–1.24)	0.113
Moderate (10–14)	**1.20**	(1.02–1.39)	0.024	**1.20**	(1.03–1.40)	0.020	**1.22**	(1.01–1.47)	0.043	1.16	(0.96–1.40)	0.133
Moderately severe (15–19)	**1.30**	(1.05–1.62)	0.018	**1.48**	(1.19–1.83)	< 0.001	**1.38**	(1.04–1.82)	0.025	**1.46**	(1.11–1.93)	0.007
Severe (20–27)	**1.46**	(1.07–2.00)	0.017	**1.69**	(1.26–2.25)	< 0.001	**1.63**	(1.08–2.47)	0.020	**1.60**	(1.07–2.40)	0.022
Generalized anxiety (GAD-7)												
Minimal (0–4)	reference			reference			reference			reference		
Mild (5–9)	**1.13**	(1.00–1.27)	0.045	**1.18**	(1.05–1.32)	0.005	1.00	(0.87–1.16)	0.965	1.08	(0.94–1.24)	0.291
Moderate (10–14)	1.16	(0.97–1.39)	0.101	1.14	(0.94–1.37)	0.181	0.96	(0.75–1.21)	0.706	0.94	(0.74–1.20)	0.629
Severe (15–21)	1.17	(0.90–1.51)	0.243	**1.51**	(1.20–1.91)	< 0.001	0.86	(0.60–1.23)	0.402	1.10	(0.77–1.55)	0.606
Fear of COVID-19 (FCV-19S)												
No fear (7–15)	reference			reference			reference			reference		
Mild (16–20)	**0.88**	(0.79–0.98)	0.020	**0.89**	(0.79–1.00)	0.043	**0.86**	(0.77–0.96)	0.006	**0.86**	(0.76–0.96)	0.009
Moderate (21–25)	0.96	(0.85–1.08)	0.482	0.96	(0.85–1.08)	0.466	0.90	(0.80–1.02)	0.094	**0.88**	(0.78–1.00)	0.046
Severe (26–35)	**0.81**	(0.69–0.95)	0.008	**0.81**	(0.70–0.95)	0.007	**0.74**	(0.62–0.87)	< 0.001	**0.70**	(0.59–0.82)	< 0.001
Unwilling to vaccinate at wave 3												
Generalized trust												
Most people can be trusted	reference			reference			reference			reference		
Need to be very careful	**1.58**	((1.34–1.85)	< 0.001	**1.58**	(1.34–1.86)	< 0.001	**1.59**	(1.35–1.87)	< 0.001	**1.63**	(1.38–1.93)	< 0.001
Don’t know	**1.84**	(1.33–2.54)	< 0.001	**1.76**	(1.28–2.43)	< 0.001	**1.90**	(1.37–2.63)	< 0.001	**1.95**	(1.41–2.70)	< 0.001
Depression (PHQ-9)												
None (0–4)	reference			reference			reference			reference		
Mild (5–9)	**1.26**	(1.07–1.48)	0.005	1.09	(0.92–1.29)	0.307	**1.30**	(1.08–1.55)	0.005	1.14	(0.94–1.38)	0.191
Moderate (10–14)	1.18	(0.93–1.50)	0.162	**1.50**	(1.20–1.88)	< 0.001	1.19	(0.89–1.59)	0.253	**1.41**	(1.06–1.88)	0.020
Moderately severe (15–19)	**1.61**	(1.19–2.17)	0.002	**1.83**	(1.36–2.47)	< 0.001	1.48	(0.99–2.22)	0.055	1.44	(0.96–2.16)	0.074
Severe (20–27)	**1.84**	(1.21–2.79)	0.004	**1.97**	(1.34–2.89)	< 0.001	1.53	(0.87–2.71)	0.140	1.29	(0.74–2.25)	0.376
Generalized anxiety (GAD-7)												
Minimal (0–4)	reference			reference			reference			reference		
Mild (5–9)	1.08	(0.90–1.29)	0.433	1.15	(0.96–1.37)	0.132	1.03	(0.83–1.29)	0.765	1.18	(0.95–1.48)	0.141
Moderate (10–14)	**1.36**	(1.05–1.76)	0.019	**1.56**	(1.21–2.02)	< 0.001	1.24	(0.88–1.75)	0.228	**1.56**	(1.10–2.21)	0.014
Severe (15–21)	**1.54**	(1.09–2.17)	0.014	**2.02**	(1.48–2.75)	< 0.001	1.28	(0.78–2.10)	0.331	**2.01**	(1.24–3.25)	0.005
Fear of COVID-19 (FCV-19S)												
No fear (7–15)	reference			reference			reference			reference		
Mild (16–20)	**0.52**	(0.44–0.61)	< 0.001	**0.37**	(0.31–0.44)	< 0.001	**0.50**	(0.42–0.59)	< 0.001	**0.35**	(0.29–0.42)	< 0.001
Moderate (21–25)	**0.52**	(0.43–0.62)	< 0.001	**0.38**	(0.32–0.46)	< 0.001	**0.47**	(0.39–0.56)	< 0.001	**0.33**	(0.27–0.39)	< 0.001
Severe (26–35)	**0.50**	(0.39–0.64)	< 0.001	**0.32**	(0.25–0.41)	< 0.001	**0.41**	(0.31–0.53)	< 0.001	**0.22**	(0.17–0.29)	< 0.001

Boldface indicates statistical significance at the 5% level (both sides). Estimates are RRRs derived from multinomial logistic regression analyses. Model 1 adjusted for any one of generalized trust, PHQ-9, GAD-7, and FCV-19S, plus other explanatory variables (sex, age group, level of education, family members living together, employment, annual household income, bank and saving deposit amount, BMI, pre-existing conditions, and region of residence). Model 2 adjusted for all of generalized trust, PHQ-9, GAD-7, and FCV-19S together, plus other explanatory variables. For all analyses, willingness to be vaccinated at wave 3 was the outcome reference group. Generalized trust, PHQ-9, GAD-7, FCV-19S, age group, employment, annual household income, and bank and saving deposit amount in Model 1 and 2 at wave 1 and wave 3 were those at wave 1 and 3, respectively. Remaining explanatory variables were those at wave 1

*RRR* Relative Risk Ratio, *CI* Confidence Interval, *PHQ-9* Patient Health Questionnaire-9, *GAD-7* Generalized Anxiety Disorder-7, *FCV-19S* Fear of COVID-19 Scale

Regarding generalized trust, Model 1 at wave 1 and wave 3 both showed that those who answered “Need to be very careful” and “Don’t know” were more likely to be undecided, or unwilling to be vaccinated, than those who answered “Most people can be trusted.” The results were basically the same in Model 2 at wave 1 and wave 3, in which depression, generalized anxiety, and fear of COVID-19 were adjusted for.

Regarding depression, Model 1 at wave 1 and wave 3 both showed that those who were mildly depressed, moderately depressed, moderate-severely depressed, and severely depressed were more likely to be undecided than those who were not depressed. These results were basically the same in Model 2 at wave 1 and wave 3, in which generalized trust, generalized anxiety, and fear of COVID-19 were adjusted for, except that mild and moderate depression lost significance in Model 2 at wave 3. With regard to unwillingness, Model 1 at wave 1 and wave 3 showed that those who were mildly depressed (at wave 1 only), moderately depressed (at wave 3 only), moderate-severely depressed, and severely depressed were more likely to be unwilling to be vaccinated than those who were not depressed. In Model 2 at wave 1 and wave 3, in which generalized trust, generalized anxiety, and fear of COVID-19 were adjusted for, there were no significant associations between depression and unwillingness to be vaccinated except that mild depression at wave 1 and moderate depression at wave 3 remained significant.

Regarding generalized anxiety, Model 1 at wave 1 and wave 3 showed that those who were mildly anxious and severely anxious (at wave 3 only) were more likely to be undecided about whether to receive a vaccine than respondents who had minimal levels of anxiety. In Model 2 at wave 1 and wave 3, in which generalized trust, depression, and fear of COVID-19 were adjusted for, there were no significant associations between generalized anxiety and indecisiveness regarding vaccination. With regard to unwillingness, Model 1 at wave 1 and wave 3 showed that those who were moderately anxious and severely anxious were more likely to be unwilling to be vaccinated than those who had a minimal level of anxiety. In Model 2 at wave 1 and wave 3, in which generalized trust, depression, and fear of COVID-19 were adjusted for, moderate anxiety and severe anxiety at wave 1 lost significance, but those at wave 3 remained significant.

Regarding fear of COVID-19, Model 1 at wave 1 and wave 3 showed that participants with mild and severe fear of COVID-19 were less likely to be undecided regarding vaccination than those who had no fear of COVID-19. In Model 2 at wave 1 and wave 3, in which generalized trust, depression, and generalized anxiety were adjusted for, the results were basically the same except that moderate fear of COVID-19 at wave 3 gained significance. With regard to unwillingness, Model 1 at wave 1 and at wave 3 showed that respondents with mild, moderate, and severe fear of COVID-19 were less likely to be unwilling to be vaccinated than those who had no fear of COVID-19. In Model 2 at wave 1 and wave 3, in which generalized trust, depression, and fear of COVID-19 were adjusted for, the results remained basically the same.

### Association of other explanatory variables with attitudes toward vaccination against COVID-19

The results of Model 2 at wave 1, in which all explanatory variables were included, are shown in Table 3. Being female (undecided only), having a lower level of education, low household income, low savings, and low BMI were positively associated with indecisiveness or unwillingness. Older age, living only with a spouse, and having hypertension or dyslipidemia were negatively associated with indecisiveness and unwillingness. The results of Model 2 at wave 3 were basically the same (shown in Supplementary Table 1).

**Table 3:** Results of the fully adjusted multinomial logistic regression analyses at wave 1

Predictors	Undecided			Unwilling		
	RRR	95% CI	*P*-value	RRR	95% CI	*P*-value
Sex						
Male	reference			reference		
Female	**1.19**	(1.07–1.32)	0.001	1.13	(0.96–1.32)	0.145
Age group, years						
65+	reference			reference		
50–64	**2.35**	(2.04–2.70)	< 0.001	**1.82**	(1.44–2.29)	< 0.001
30–49	**3.58**	(3.06–4.19)	< 0.001	**3.04**	(2.35–3.91)	< 0.001
18–29	**4.16**	(3.35–5.16)	< 0.001	**4.46**	(3.23–6.15)	< 0.001
Highest education						
Junior/senior high school	**1.21**	(1.09–1.35)	< 0.001	**1.32**	(1.11–1.56)	0.001
Two- or three-year college	1.08	(0.96–1.22)	0.185	**1.28**	(1.06–1.54)	0.009
Four-year college or higher	reference			reference		
Family members living together						
Living alone	**1.33**	(1.15–1.54)	< 0.001	**1.46**	(1.16–1.84)	0.001
Living only with spouses	reference			reference		
Living with children	**1.15**	(1.02–1.29)	0.027	1.20	(0.98–1.47)	0.082
Living with parents	**1.34**	(1.16–1.55)	< 0.001	**1.46**	(1.16–1.84)	0.001
Three generation household	**1.49**	(1.21–1.83)	< 0.001	1.40	(0.99–1.98)	0.059
Others (siblings only, friends, grandparents and grandchildren etc.)	1.38	(0.97–1.96)	0.077	**1.88**	(1.17–3.00)	0.009
Employment						
Employed	reference			reference		
Homemaker	1.09	(0.95–1.25)	0.208	0.86	(0.68–1.08)	0.198
Not employed (seeking a job)	0.98	(0.74–1.28)	0.858	0.98	(0.67–1.45)	0.937
Not employed (not seeking job)	1.06	(0.91–1.24)	0.437	**1.29**	(1.03–1.61)	0.026
Student	0.88	(0.64–1.21)	0.420	0.72	(0.46–1.11)	0.137
Other	**1.60**	(1.05–2.42)	0.028	**1.99**	(1.14–3.47)	0.015
Annual household income, million yen						
< 3	**1.35**	(1.15–1.58)	< 0.001	**1.39**	(1.09–1.77)	0.008
3–4	0.99	(0.86–1.14)	0.871	0.96	(0.77–1.20)	0.730
5–7	1.12	(0.98–1.28)	0.086	0.87	(0.70–1.08)	0.216
≥ 8	reference			reference		
Bank and saving deposit amount, million yen						
< 1	**1.44**	(1.27–1.64)	< 0.001	**1.45**	(1.18–1.77)	< 0.001
1–3	**1.23**	(1.08–1.40)	0.002	1.13	(0.92–1.40)	0.242
4–9	**1.23**	(1.08–1.39)	0.001	1.13	(0.92–1.39)	0.238
≥ 10	reference			reference		
BMI						
< 18.5	**1.17**	(1.03–1.34)	0.019	**1.34**	(1.10–1.62)	0.003
18.5–24.9	reference			reference		
25.0-29.9	0.94	(0.83–1.07)	0.341	1.02	(0.84–1.25)	0.827
≥ 30.0	0.97	(0.75–1.26)	0.833	0.76	(0.49–1.19)	0.232
Pre-existing condition						
Hypertension	**0.79**	(0.69–0.90)	< 0.001	**0.62**	(0.49–0.78)	< 0.001
Dyslipidemia	**0.62**	(0.52–0.73)	< 0.001	**0.68**	(0.51–0.91)	0.009
Diabetes	0.95	(0.77–1.17)	0.610	0.88	(0.62–1.27)	0.501
Heart disease	0.86	(0.63–1.17)	0.338	0.94	(0.56–1.56)	0.802
Renal disease	1.12	(0.67–1.85)	0.674	0.70	(0.27–1.81)	0.467
Cancer	0.74	(0.50–1.09)	0.126	0.82	(0.44–1.52)	0.522
Respiratory disease	0.97	(0.71–1.31)	0.838	1.23	(0.78–1.92)	0.369
Other condition	1.03	(0.71–1.47)	0.890	1.02	(0.58–1.77)	0.952
Region of residence						
Tokyo	reference			reference		
Kanagawa	1.05	(0.85–1.29)	0.652	**0.64**	(0.45–0.90)	0.011
Osaka	0.93	(0.75–1.14)	0.479	0.83	(0.60–1.13)	0.237
Aichi	0.90	(0.72–1.12)	0.343	0.80	(0.57–1.11)	0.180
Saitama	1.12	(0.90–1.39)	0.319	**0.69**	(0.48–1.00)	0.049
Chiba	0.93	(0.73–1.17)	0.525	1.05	(0.75–1.47)	0.776
Hyogo	0.92	(0.72–1.17)	0.496	0.87	(0.61–1.25)	0.457
Hokkaido	0.86	(0.67–1.10)	0.225	0.80	(0.55–1.15)	0.227
Fukuoka	0.84	(0.64–1.09)	0.187	1.01	(0.70–1.46)	0.964
Tohoku region	**0.74**	(0.59–0.91)	0.005	**0.61**	(0.44–0.86)	0.004
North Kanto	1.15	(0.92–1.45)	0.228	0.84	(0.59–1.22)	0.365
Hokuriku	0.92	(0.71–1.18)	0.501	**0.64**	(0.42–0.97)	0.037
Chubu	1.06	(0.86–1.30)	0.593	**0.67**	(0.47–0.93)	0.019
Kinki	0.96	(0.78–1.19)	0.713	0.96	(0.71–1.31)	0.817
Chugoku	**1.30**	(1.05–1.63)	0.018	0.89	(0.63–1.27)	0.535
Shikoku	0.81	(0.60–1.09)	0.156	0.81	(0.52–1.27)	0.358
Kyushu and Okinawa	1.10	(0.89–1.36)	0.389	0.86	(0.61–1.19)	0.354
Generalized trust						
Most people can be trusted	reference			reference		
Need to be very careful	**1.28**	(1.16–1.41)	< 0.001	**1.59**	(1.35–1.87)	< 0.001
Don’t know	**1.65**	(1.34–2.04)	< 0.001	**1.90**	(1.37–2.63)	< 0.001
Depression (PHQ-9)						
None (0–4)	reference			reference		
Mild (5–9)	**1.19**	(1.06–1.34)	0.003	**1.30**	(1.08–1.55)	0.005
Moderate (10–14)	**1.22**	(1.01–1.47)	0.043	1.19	(0.89–1.59)	0.253
Moderately severe (15–-19)	**1.38**	(1.04–1.82)	0.025	1.48	(0.99–2.22)	0.055
Severe (20–27)	**1.63**	(1.08–2.47)	0.020	1.53	(0.87–2.71)	0.140
Generalized anxiety (GAD-7)						
Minimal (0–4)	reference			reference		
Mild (5–9)	1.00	(0.87–1.16)	0.965	1.03	(0.83–1.29)	0.765
Moderate (10–14)	0.96	(0.75–1.21)	0.706	1.24	(0.88–1.75)	0.228
Severe (15–21)	0.86	(0.60–1.23)	0.402	1.28	(0.78–2.10)	0.331
Fear of COVID-19 (FCV-19S)						
No fear (7–15)	reference			reference		
Mild (16–20)	**0.86**	(0.77–0.96)	0.006	**0.50**	(0.42–0.59)	< 0.001
Moderate (21–25)	0.90	(0.80–1.02)	0.094	**0.47**	(0.39–0.56)	< 0.001
Severe (26–35)	**0.74**	(0.62–0.87)	< 0.001	**0.41**	(0.31–0.53)	< 0.001

Boldface indicates statistical significance at the 5% level (both sides). Estimates are RRRs derived from multinomial logistic regression analyses adjusting for all of generalized trust, PHQ-9, GAD-7, and FCV-19S, plus other explanatory variables (sex, age group, level of education, family members living together, employment, annual household income, bank and saving deposit amount, BMI, pre-existing conditions, and region of residence). All explanatory variables are those at wave 1. Willingness to be vaccinated at wave 3 was the outcome reference group

*RRR* Relative Risk Ratio, *CI* Confidence Interval, *PHQ-9* Patient Health Questionnaire-9, *GAD-7* Generalized Anxiety Disorder −7, *FCV-19S* Fear of COVID-19 Scale

## Discussion

In the present study, we explored whether generalized trust, mental health factors such as depression and generalized anxiety, and fear of COVID-19 are predictors of vaccine hesitancy against COVID-19. We used the data on wave 1 (between October 27 and November 6, 2020) and wave 3 (between April 23 and May 6, 2021) of a longitudinal online survey carried out in Japan. We found that: (1) respondents who had lower levels of generalized trust at wave 1 and 3 were more likely to be undecided or unwilling to be vaccinated at wave 3; (2) respondents with moderately severe or severe levels of depression at wave 1 and 3 were more likely to be undecided about being vaccinated at wave 3; (3) respondents with moderate or severe levels of generalized anxiety at wave 3 but not at wave 1 were more likely to be unwilling to be vaccinated at wave 3; and (4) participants with high levels of fear of COVID-19 at wave 1 and 3 were less likely to be undecided or unwilling to be vaccinated at wave 3. In addition to these results, our findings confirmed the results of previous studies that found that younger people, women, people with low incomes, and people with lower levels of education were more likely to be undecided or unwilling to be vaccinated.

The wave 3 survey, in which the question on vaccine hesitancy was asked, was conducted between April 23 and May 6, 2021, in Japan, where vaccination against COVID-19 for medical personnel began on February 17, 2021, a few months later than Europe and the United States. On April 12, vaccination for persons aged 65 and over was initiated. As of April 23, the shares of the Japanese populations that had received at least one dose of the COVID-19 vaccine and been fully vaccinated against COVID-19 were 1.7 and 0.7%, respectively [1]. As of April 23, total cases of COVID-19 per million people were 4426 and total deaths per million people were 78 in Japan [1]. Hence, the ratios of those who were vaccinated against COVID-19 and casualties due to COVID-19 were lower in Japan than in many other countries. The declaration of a state of emergency was in effect in several areas of Japan including Tokyo and Osaka at that time in order to inhibit the spread of COVID-19. The results of the present study must be interpreted against this background.

Regarding generalized trust in people, the present study showed that participants with low levels of generalized trust at waves 1 and 3 were more likely to be undecided or unwilling to be vaccinated at wave 3. Wave 1 was approximately 6 months before wave 3. Therefore, the present study suggest that the level of generalized trust predicts vaccine hesitancy 6 months later. This finding is consistent with the two previous studies that reported that those who indicated a higher level of generalized trust tended to be more willing to get a COVID-19 vaccine than those with a lower level of generalized trust [31, 36]. The relationship between generalized trust and vaccine hesitancy in general was addressed in a systematic review by Larson et al. [25]. However, only three of the reviewed studies examined the relationship between generalized trust and vaccination, and one of them examined trust in the government. Two studies [37, 39] referred to in Larson et al. and another study [38] reported findings consistent with the present study; that is, participants with high generalized trust were more willing to be vaccinated against influenza. Hence, studies thus far, including the present one, have consistently shown that generalized trust is positively associated with vaccine acceptance. How generalized trust is related to vaccine acceptance not only with regard to COVID 19, but also other diseases, has not been sufficiently explored. Although we did not examine the mechanisms linking generalized trust and vaccine acceptance in the present study, previous studies suggest two possible explanations. First, individuals with high levels of generalized trust tend to be more altruistic and intend to contribute to the public health policy of containing COVID-19 [37]. Another possible explanation is that individuals with lower levels of generalized trust may have distrust related to a wide range of issues, including the performance of government [65, 66], the safety of the vaccine, and even medical treatments in general [31], leading to vaccine hesitancy. The association between vaccine acceptance and various forms of specific trust, including trust in vaccines, trust in the government, trust in the health system, or in professionals, has already been shown in COVID-19 vaccination [6, 16, 20, 22, 26–33] and in general [24, 25]. Several studies, mainly in the social sciences, have demonstrated the positive association between generalized trust and some types of specific trust [65, 66]. However, to our knowledge, the mediating role of specific trust between generalized trust and vaccine hesitancy has only been explored in Ahorsu et al. [31]. Further studies are recommended to explore how lower levels of generalized trust are associated with COVID-19 vaccine hesitancy.

Regarding the relationship between depression/anxiety and attitudes toward vaccination, the present study showed that participants with depression at waves 1 and 3 were more likely to be undecided regarding vaccination at wave 3, and participants with generalized anxiety at wave 3 but not at wave 1 were more likely to be unwilling to be vaccinated at wave 3. There are two things to be considered regarding this finding based on previous studies dealing with similar topics. First, studies in the United States, China, and Germany [15, 17, 41] found no significant association between depression/anxiety and attitudes toward COVID-19 vaccination, whereas the present study as well as some previous studies [22, 42] found a positive association between psychological distress and vaccine hesitancy. The reason for this disparity is unclear but might be due to the large sample size of our study, which enabled the identification of even minor differences. Another thing to be considered is that studies that address the relationship between depression/anxiety and attitudes toward vaccination other than COVID-19 [43–45] seem to have contradictory results compared to the present study and the two above-mentioned studies on COVID-19 [22, 42], despite the fact that the themes these studies addressed are mostly the same. Chan et al. [43] showed that those who were more anxious were more willing to receive vaccination against A/H7N9 influenza in Hong Kong. Lawrence et al. [44] reported that older primary care patients with mental health diagnoses were more likely to receive vaccination against influenza although the outcome in this study was real vaccine uptake instead of willingness to receive vaccination. Similarly, Mohammed et al. [45] reported that pregnant women with mild depression/anxiety were more likely to receive influenza vaccination during pregnancy although there was no association between depression/anxiety and willingness to receive vaccination.

Questions still remain as to how mental health factors such as depression and anxiety are associated with vaccine hesitancy in some studies regarding COVID-19, and why the studies on influenza seem to have contradictory results. In addressing these questions, the differences between depression and anxiety may need to be considered. One characteristic of people with depression is that they tend to have difficulty making decisions [67]. The present study’s finding that people with depression tend to be undecided about COVID-19 vaccination may be explained by this general indecisiveness. People with generalized anxiety are generally risk averse [68], but what they are anxious about is not predetermined. In the case of vaccination, while there are risks such as side effects from the vaccine, there is also an increased risk of becoming infected with COVID-19 and severely ill by not getting vaccinated. Hence, which of these two risks people with generalized anxiety will prioritize is difficult to determine beforehand without data analysis. The results of the present study suggest that those with moderate and severe level of generalized anxiety at the moment tend to prioritize avoiding the risks associated with getting vaccinated against COVID-19 rather than being infected with COVID-19. Contrastingly, the results of the previous studies [43–45] suggest that those with moderate and severe levels of generalized anxiety tend to prioritize the avoidance of the risks associated with infection (mainly for influenza) over the risks associated with the vaccine. Although we do not have clear answers to explain this seeming difference, there are several possible explanations. First, the subjects in Lawrence et al. [44] were older primary care patients and the subjects in Mohammed et al. [45] were pregnant women who attended an antenatal clinic. By contrast, the subjects in the present study were mostly members of the general public and did not belong in a clinical setting. This difference may explain the variation in results. Second, Chan et al. [43] demonstrated a positive association between anxiety level and willingness to be vaccinated for A/H7N9 influenza based on a population telephone survey, whereas the present study examined the willingness to receive vaccination against COVID-19 based on an online survey. Such differences may explain the seemingly contradictory results. Overall, studies exploring the relationship between depression/anxiety and vaccine hesitancy have shown varied results. The diseases that the vaccines are meant to prevent (e.g., COVID-19, seasonal influenza), the methods of measurement of depression/anxiety (e.g., self-administered questionnaire, diagnoses at clinical settings), the coverage of study subjects (e.g., elderly people, pregnant women, general population), as well as the differences in cultural and political backgrounds may be responsible for the varied results in different studies. Further studies are recommended, considering the results of previous studies that are inconsistent with the present study [15, 17, 41, 43–45] and actual vaccination behaviors rather than willingness to receive a vaccine.

The present study found that people with high levels of fear of COVID-19 at waves 1 and 3 were more likely to be willing to receive a vaccine at wave 3. This finding is consistent with most studies that addressed this issue [15, 22, 26, 27, 41, 46–48, 54]. A typical characteristic of fear is that people tend to avoid what they fear. Hence, the most plausible explanation of the negative association between the fear of COVID-19 and vaccine hesitancy is that people with high levels of fear of COVID-19 try to avoid what they fear, namely COVID-19, by getting vaccinated against it. In contrast to most studies including the present one, an older study carried out in Lebanon [46] found no significant association between the willingness to receive the COVID-19 vaccine and the fear of COVID-19. The reasons for the difference might be cultural or a difference in sample size.

According to the present study, younger people, women, people with low incomes, and people with lower levels of education are more likely to be undecided or unwilling to be vaccinated. These results are consistent with many previous studies that claim that younger people [6, 7, 11, 13–15, 18–22], women [6, 7, 9–12, 14–16, 19–23], people with low incomes [6, 11, 13–15, 18, 19, 21, 22], and people with lower levels of education [6, 7, 10, 13–16, 20, 22, 23] were reluctant to receive a vaccine. Vaccine hesitancy due to younger age and being female may be due to decreased anxiety compared to older males, as there is widely shared information that older people and men are more likely to be seriously affected by COVID-19 infection [69, 70]. Regarding socioeconomic status derived mainly based on income and education, a plausible mediator between low socioeconomic status and vaccine hesitancy is low levels of health protective behaviors due to poor health literacy in people with low socioeconomic status [71–73]. A mediating role of health literacy between socioeconomic status and COVID-19 vaccination hesitancy is suggested in a previous article [72], although it is unclear whether health literacy is the only mediator between them [74]. Therefore, it is safe to state that mechanisms linking such sociodemographic variables and vaccine hesitancy have not been sufficiently explored so far, and further investigation is recommended.

The following limitations must be kept in mind when considering the results of the present study. First, the participants in the present study were not representative of the general population. The present study was based on wave 1 and wave 3 of a longitudinal online survey with the respondents limited to those who responded both at wave 1 and wave 3. Although wave 1 was designed to reflect sex, age, and place of residence at the prefecture level of the entire Japanese population, there was no random sampling in the extraction of the survey participants. In addition, the number of valid respondents at wave 3 was approximately 70% of wave 1. Moreover, the present study was an Internet-based survey; participants were required to have access to the Internet. Possibly reflecting this requirement, almost half of our participants had attended four-year colleges, a percentage greater than Japan’s national average. Second, potentially important confounders may not have been adjusted for in the present study. We asked only one question on generalized trust. Specific trusts not covered by the present study may be confounders. Other potential confounders such as place of residence (rural vs urban area) were not incorporated in the present study due to the data constraints. Hence, there remains a possibility that associations between generalized trust (or depression/anxiety, fear of COVID-19) and vaccine hesitancy might be spurious. Third, unlike in many other studies on vaccine hesitancy, COVID-19 vaccination had already started when the question on vaccination was being asked (wave 3), although less than 2% of Japanese people had been vaccinated by wave 3. This may have had some impact on attitudes toward vaccine hesitancy. Finally, the present study was an observational study and, thus, cannot demonstrate a causal relationship between the explanatory variables and the outcome variables.

## Conclusions

The present study explored the associations between unwillingness and indecisiveness regarding being vaccinated against COVID-19 with generalized trust, depression, and generalized anxiety. In our findings, low generalized trust, depression, and generalized anxiety were associated with unwillingness and indecisiveness to be vaccinated against COVID-19. In order to determine whether such associations are observed in other countries with different cultures, further epidemiological or sociological studies using global data may be needed.

## Supplementary Information

**Additional file 1: Supplementary Table 1.** Results of the Fully Adjusted Multinomial Logistic Regression Analyses at wave 3.

## Data Availability

The data used in the present study belong to the RIETI and can be obtained from the institute upon reasonable request.

## Abbreviations

BMI: Body mass index
COVID-19: Coronavirus disease of 2019
FCV-19S: Fear of COVID-19 Scale
GAD-7: Generalized Anxiety Disorder-7
PHQ-9: Patient Health Questionnaire-9
RIETI: Research Institute of Economy, Trade and Industry
RRR: Relative risk ratio
WVS: World Values Survey
