# Supplementary material for: Association between COVID-19 vaccine hesitancy and generalized trust, depression, generalized anxiety, and fear of COVID-19
Source: BMC Public Health. 2022 Jan 18;22:126. doi: 10.1186/s12889-021-12479-w (PMC8764499; doi:10.1186/s12889-021-12479-w)
Supplement: Supplementary file 1 — Additional file 1: Supplementary Table 1. Results of the Fully Adjusted Multinomial Logistic Regression Analyses at wave 3. [file 12889_2021_12479_MOESM1_ESM.docx]

Supplementary Table 1. Results of the Fully Adjusted Multinomial Logistic Regression Analyses at wave 3

| Predictors |  | Undecided | | | | Unwilling | | | |
| --- | --- | --- | --- | --- | --- | --- | --- | --- | --- |
|  |  | RRR | 95% CI | | *P*-value | RRR | 95% CI | | *P*-value |
| Sex | Male | reference | | |  | reference | | |  |
|  | Female | **1.22** | (1.10 - | 1.36) | <0.001 | **1.23** | (1.05 - | 1.45) | 0.011 |
| Age group, years | 65+ | reference | | |  | reference | | |  |
|  | 50–64 | **2.32** | (2.02 - | 2.66) | <0.001 | **1.88** | (1.49 - | 2.36) | <0.001 |
|  | 30–49 | **3.41** | (2.92 - | 3.98) | <0.001 | **2.92** | (2.27 - | 3.75) | <0.001 |
|  | 18–29 | **3.90** | (3.13 - | 4.86) | <0.001 | **4.45** | (3.22 - | 6.16) | <0.001 |
| Highest education | Junior/senior high school | **1.20** | (1.08 - | 1.34) | <0.001 | **1.31** | (1.11 - | 1.56) | 0.002 |
|  | Two- or three-year college | 1.08 | (0.96 - | 1.21) | 0.225 | **1.28** | (1.06 - | 1.55) | 0.009 |
|  | Four-year college or higher | reference | | |  | reference | | |  |
| Family members living together | Living alone | **1.33** | (1.15 - | 1.54) | <0.001 | **1.44** | (1.14 - | 1.81) | 0.002 |
|  | Living only with spouses | reference | | |  | reference | | |  |
|  | Living with children | **1.15** | (1.02 - | 1.30) | 0.020 | 1.20 | (0.98 - | 1.47) | 0.084 |
|  | Living with parents | **1.36** | (1.17 - | 1.57) | <0.001 | **1.42** | (1.13 - | 1.80) | 0.003 |
|  | Three generation household | **1.50** | (1.22 - | 1.85) | <0.001 | 1.32 | (0.93 - | 1.87) | 0.126 |
|  | Others (siblings only, friends, grandparents and grandchildren etc.） | 1.34 | (0.94 - | 1.91) | 0.106 | **1.72** | (1.07 - | 2.77) | 0.025 |
| Employment | Employed | reference | | |  | reference | | |  |
|  | Homemaker | 1.07 | (0.93 - | 1.22) | 0.326 | 0.83 | (0.66 - | 1.05) | 0.130 |
|  | Not employed (seeking a job） | 0.86 | (0.65 - | 1.13) | 0.276 | 1.26 | (0.88 - | 1.81) | 0.210 |
|  | Not employed (not seeking job) | 1.04 | (0.89 - | 1.20) | 0.653 | **1.36** | (1.09 - | 1.69) | 0.007 |
|  | Student | 0.80 | (0.57 - | 1.13) | 0.212 | **0.61** | (0.38 - | 0.99) | 0.047 |
|  | Other | **1.52** | (1.00 - | 2.29) | 0.047 | **2.28** | (1.34 - | 3.87) | 0.002 |
| Annual household income, million yen | < 3 | **1.32** | (1.13 - | 1.55) | <0.001 | 1.23 | (0.96 - | 1.57) | 0.096 |
|  | 3–4 | 1.06 | (0.92 - | 1.22) | 0.427 | 0.93 | (0.74 - | 1.17) | 0.543 |
|  | 5–7 | 1.14 | (1.00 - | 1.30) | 0.053 | 0.83 | (0.67 - | 1.04) | 0.099 |
|  | ≥ 8 | reference | | |  | reference | | |  |
| Bank and saving deposit amount, million yen | < 1 | **1.48** | (1.30 - | 1.68) | <0.001 | **1.51** | (1.24 - | 1.84) | <0.001 |
|  | 1–3 | **1.23** | (1.07 - | 1.40) | 0.002 | 1.05 | (0.84 - | 1.30) | 0.672 |
|  | 4–9 | **1.19** | (1.05 - | 1.35) | 0.006 | 1.01 | (0.82 - | 1.24) | 0.947 |
|  | ≥ 10 | reference | | |  | reference | | |  |
| BMI | < 18.5 | **1.17** | (1.02 - | 1.33) | 0.022 | **1.34** | (1.11 - | 1.64) | 0.003 |
|  | 18.5–24.9 | reference | | |  | reference | | |  |
|  | 25.0-29.9 | 0.94 | (0.83 - | 1.06) | 0.318 | 1.01 | (0.83 - | 1.24) | 0.897 |
|  | ≥30.0 | 0.96 | (0.75 - | 1.25) | 0.778 | 0.78 | (0.50 - | 1.22) | 0.274 |
| Pre-existing condition | Hypertension | **0.78** | (0.69 - | 0.90) | <0.001 | **0.62** | (0.49 - | 0.79) | <0.001 |
|  | Dyslipidemia | **0.62** | (0.52 - | 0.73) | <0.001 | **0.69** | (0.52 - | 0.92) | 0.013 |
|  | Diabetes | 0.95 | (0.77 - | 1.18) | 0.664 | 0.92 | (0.64 - | 1.32) | 0.647 |
|  | Heart disease | 0.85 | (0.62 - | 1.17) | 0.314 | 0.94 | (0.56 - | 1.57) | 0.814 |
|  | Renal disease | 1.09 | (0.65 - | 1.81) | 0.751 | 0.69 | (0.27 - | 1.79) | 0.451 |
|  | Cancer | 0.78 | (0.53 - | 1.16) | 0.222 | 0.88 | (0.47 - | 1.65) | 0.699 |
|  | Respiratory disease | 0.95 | (0.70 - | 1.29) | 0.737 | 1.25 | (0.79 - | 1.95) | 0.339 |
|  | Other condition | 1.02 | (0.71 - | 1.46) | 0.936 | 1.02 | (0.58 - | 1.79) | 0.940 |
| Region of residence | Tokyo | reference | | |  | reference | | |  |
|  | Kanagawa | 1.05 | (0.86 - | 1.29) | 0.636 | **0.63** | (0.44 - | 0.89) | 0.008 |
|  | Osaka | 0.92 | (0.75 - | 1.14) | 0.465 | 0.83 | (0.60 - | 1.14) | 0.246 |
|  | Aichi | 0.89 | (0.71 - | 1.11) | 0.284 | 0.79 | (0.56 - | 1.11) | 0.172 |
|  | Saitama | 1.11 | (0.89 - | 1.38) | 0.355 | **0.68** | (0.47 - | 0.99) | 0.043 |
|  | Chiba | 0.92 | (0.73 - | 1.17) | 0.496 | 1.07 | (0.76 - | 1.50) | 0.704 |
|  | Hyogo | 0.93 | (0.73 - | 1.19) | 0.560 | 0.93 | (0.64 - | 1.34) | 0.689 |
|  | Hokkaido | 0.85 | (0.66 - | 1.09) | 0.196 | 0.79 | (0.54 - | 1.15) | 0.221 |
|  | Fukuoka | 0.86 | (0.66 - | 1.12) | 0.275 | 1.05 | (0.72 - | 1.52) | 0.814 |
|  | Tohoku region | **0.73** | (0.59 - | 0.90) | 0.004 | **0.62** | (0.44 - | 0.87) | 0.005 |
|  | North Kanto | 1.17 | (0.93 - | 1.47) | 0.186 | 0.89 | (0.61 - | 1.28) | 0.520 |
|  | Hokuriku | 0.91 | (0.71 - | 1.18) | 0.487 | **0.62** | (0.41 - | 0.95) | 0.026 |
|  | Chubu | 1.08 | (0.87 - | 1.33) | 0.494 | **0.69** | (0.49 - | 0.98) | 0.036 |
|  | Kinki | 0.97 | (0.78 - | 1.19) | 0.752 | 0.99 | (0.72 - | 1.36) | 0.955 |
|  | Chugoku | **1.30** | (1.05 - | 1.63) | 0.018 | 0.93 | (0.65 - | 1.33) | 0.700 |
|  | Shikoku | 0.80 | (0.59 - | 1.07) | 0.138 | 0.81 | (0.52 - | 1.27) | 0.357 |
|  | Kyushu and Okinawa | 1.10 | (0.89 - | 1.37) | 0.390 | 0.86 | (0.62 - | 1.21) | 0.392 |
| Generalized trust | Most people can be trusted | reference | | |  | reference | | |  |
|  | Need to be very careful | **1.45** | (1.32 - | 1.61) | <0.001 | **1.63** | (1.38 - | 1.93) | <0.001 |
|  | Don’t know | **1.75** | (1.42 - | 2.16) | <0.001 | **1.95** | (1.41 - | 2.70) | <0.001 |
| Depression  (PHQ-9) | None (0–4) | reference | | |  | reference | | |  |
|  | Mild (5–9) | 1.10 | (0.98 - | 1.24) | 0.113 | 1.14 | (0.94 - | 1.38) | 0.191 |
|  | Moderate (10–14) | 1.16 | (0.96 - | 1.40) | 0.133 | **1.41** | (1.06 - | 1.88) | 0.020 |
|  | Moderately severe (15–-19) | **1.46** | (1.11 - | 1.93) | 0.007 | 1.44 | (0.96 - | 2.16) | 0.074 |
|  | Severe (20–27) | **1.60** | (1.07 - | 2.40) | 0.022 | 1.29 | (0.74 - | 2.25) | 0.376 |
| Generalized anxiety  (GAD-7) | Minimal (0–4) | reference | | |  | reference | | |  |
|  | Mild (5–9) | 1.08 | (0.94 - | 1.24) | 0.291 | 1.18 | (0.95 - | 1.48) | 0.141 |
|  | Moderate (10–14) | 0.94 | (0.74 - | 1.20) | 0.629 | **1.56** | (1.10 - | 2.21) | 0.014 |
|  | Severe (15–21) | 1.10 | (0.77 - | 1.55) | 0.606 | **2.01** | (1.24 - | 3.25) | 0.005 |
| Fear of  COVID-19  (FCV-19S) | No fear (7–15) | reference | | |  | reference | | |  |
|  | Mild (16–20) | **0.86** | (0.76 - | 0.96) | 0.009 | **0.35** | (0.29 - | 0.42) | <0.001 |
|  | Moderate (21–25) | **0.88** | (0.78 - | 1.00) | 0.046 | **0.33** | (0.27 - | 0.39) | <0.001 |
|  | Severe (26–35) | **0.70** | (0.59 - | 0.82) | <0.001 | **0.22** | (0.17 - | 0.29) | <0.001 |

Note: Boldface indicates statistical significance at the 5% level (both sides). RRR = Relative Risk Ratio; CI = Confidence Interval; PHQ-9 = Patient Health Questionnaire-9; GAD-7 = Generalized Anxiety Disorder -7; FCV-19S = Fear of COVID-19 Scale. Estimates are RRRs derived from multinomial logistic regression analyses adjusting for all of generalized trust, PHQ-9, GAD-7, and FCV-19S, plus other explanatory variables (sex, age group, level of education, family members living together, employment, annual household income, bank and saving deposit amount, BMI, pre-existing conditions, and region of residence). Generalized trust, PHQ-9, GAD-7, FCV-19S, age group, employment, annual household income, and bank and saving deposit amounts are those at wave 3. Remaining explanatory variables were those at wave 1. Willingness to be vaccinated at wave 3 was the outcome reference group.
